# Efficacy of RTS,S malaria vaccines: individual-participant pooled analysis of phase 2 data

**DOI:** 10.1016/S1473-3099(13)70005-7

**Published:** 2013-01

**Authors:** Philip Bejon, Michael T White, Ally Olotu, Kalifa Bojang, John PA Lusingu, Nahya Salim, Nekoye N Otsyula, Selidji T Agnandji, Kwaku Poku Asante, Seth Owusu-Agyei, Salim Abdulla, Azra C Ghani

**Affiliations:** a KEMRI-Wellcome Trust Research Programme, Kenya Medical Research Institute, Kilifi, Kenya; b Centre for Clinical Vaccinology and Tropical Medicine, University of Oxford, UK; c MRC Centre for Outbreak Analysis and Modelling, Imperial College London, London, UK; d Medical Research Council Laboratories, Fajara, The Gambia; e National Institute for Medical Research, Tanga Centre, Tanga, Tanzania; f Ifakara Health Institute, Bagamoyo, Tanzania; g Kenya Medical Research Institute, and US Army Medical Research Unit–Kenya, Nairobi, Kenya; h Medical Research Unit, Albert Schweitzer Hospital, Lambaréné, Gabon; i Kintampo Health Research Centre, Kintampo, Ghana

## Abstract

**Background:**

The efficacy of RTS,S/AS01 as a vaccine for malaria is being tested in a phase 3 clinical trial. Early results show significant, albeit partial, protection against clinical malaria and severe malaria. To ascertain variations in vaccine efficacy according to covariates such as transmission intensity, choice of adjuvant, age at vaccination, and bednet use, we did an individual-participant pooled analysis of phase 2 clinical data.

**Methods:**

We analysed data from 11 different sites in Africa, including 4453 participants. We measured heterogeneity in vaccine efficacy by estimating the interactions between covariates and vaccination in pooled multivariable Cox regression and Poisson regression analyses. Endpoints for measurement of vaccine efficacy were infection, clinical malaria, severe malaria, and death. We defined transmission intensity levels according to the estimated local parasite prevalence in children aged 2–10 years (PrP_2–10_), ranging from 5% to 80%. Choice of adjuvant was either AS01 or AS02.

**Findings:**

Vaccine efficacy against all episodes of clinical malaria varied by transmission intensity (p=0·001). At low transmission (PrP_2–10_ 10%) vaccine efficacy was 60% (95% CI 54 to 67), at moderate transmission (PrP_2–10_ 20%) it was 41% (21 to 57), and at high transmission (PrP_2–10_ 70%) the efficacy was 4% (−10 to 22). Vaccine efficacy also varied by adjuvant choice (p<0·0001)—eg, at low transmission (PrP_2–10_ 10%), efficacy varied from 60% (95% CI 54 to 67) for AS01 to 47% (14 to 75) for AS02. Variations in efficacy by age at vaccination were of borderline significance (p=0·038), and bednet use and sex were not significant covariates. Vaccine efficacy (pooled across adjuvant choice and transmission intensity) varied significantly (p<0·0001) according to time since vaccination, from 36% efficacy (95% CI 24 to 45) at time of vaccination to 0% (−38 to 38) after 3 years.

**Interpretation:**

Vaccine efficacy against clinical disease was of limited duration and was not detectable 3 years after vaccination. Furthermore, efficacy fell with increasing transmission intensity. Outcomes after vaccination cannot be gauged accurately on the basis of one pooled efficacy figure. However, predictions of public-health outcomes of vaccination will need to take account of variations in efficacy by transmission intensity and by time since vaccination.

**Funding:**

Medical Research Council (UK); Bill & Melinda Gates Foundation Vaccine Modelling Initiative; Wellcome Trust.

## Introduction

The increasing application of interventions for malaria control over the past 10 years has been linked to reductions in morbidity and mortality associated with malaria infection.^1^, ^2^ A vaccine for malaria could have an important role in further reduction of the burden of disease. The candidate malaria vaccine RTS,S/AS01 is now in phase 3 clinical trials, for which preliminary data for the first 12 months of follow-up are available.^3^ Efficacy against clinical malaria was 55·8% (97·5% CI 50·6–60·4) among children age 5–17 months. Combined efficacy against severe malaria for children aged 5–17 months and 6–12 weeks was 34·8% (95% CI 16·2–49·2).

RTS,S protects at pre-erythrocytic stages of the parasite's lifecycle. It is partly effective and has been described as a leaky vaccine^4^—ie, no individual is protected consistently against every episode of exposure, but the risk of acquiring infection after any single episode of exposure is reduced. In field trials, RTS,S has been given with either of two different adjuvant systems: AS01 or AS02. Although RTS,S/AS01 seems to be more immunogenic than RTS,S/AS02, efficacy trials of RTS,S/AS01 and RTS,S/AS02 have not resulted in definitively powered comparisons.^5^, ^6^ Furthermore, the variation in vaccine efficacy over time remains unknown, with conflicting evidence from individual trials.

Vaccine efficacies are usually summarised with point estimates. However, if vaccine efficacy is heterogeneous by subgroups within the population, this efficacy figure will be a mean of the vaccine efficacy in the various subgroups, weighted according to the proportion of the population.^7^ For instance, if vaccine efficacy is higher in older children then the overall efficacy in a particular trial will depend on the proportion of older children to younger children that are vaccinated.

Analysis of phase 2b data to date shows variations in measured efficacy between trials.^6^, ^8^, ^9^, ^10^, ^11^, ^12^, ^13^, ^14^, ^15^, ^16^ These differences might be attributable to the vaccine formulation, intensity of transmission, length of follow-up, or age-range of participants. To ascertain which covariates are associated with variations in vaccine efficacy, we did a pooled analysis of data from phase 2b trials.

## Methods

### Data collection

We identified phase 2b trials^6^, ^8^, ^9^, ^10^, ^11^, ^12^, ^13^, ^14^, ^15^, ^16^ of RTS,S from the GlaxoSmithKline Biologicals registry of trials (data on file), and raw data were provided by GSK Biologicals to three academic investigators (PB, MTW, and ACG). One of us (PB) checked data for completeness by comparing data summaries with the primary publications; all investigators analysed the data. Characteristics of the trials, done at 11 sites in total (from six countries), are summarised in table 1.

**Table 1:** Characteristics of different sites

	**Patients (n)**	**Active vaccine**	**Control vaccine**	**Surveillance**	**Median age (IQR)**	Local parasite prevalence (%)*	**Bednet use**	**Efficacy (95% CI)**
Gambia^8^	250	RTS,S/AS02A	Rabies	ACDi, weekly blood films	24 years (19–34)	70	13%	34% (8 to 53)
Mozambique (cohort 1)^10^, ^14^, ^16^	1589	RTS,S/AS02A	Hepatitis B or pneumococcal conjugate/*Haemophilus influenzae* type B	PCD	35 months (24–48)	40	4·5%	30% (11 to 45)
Mozambique (cohort 2)^10^, ^14^, ^16^	411	RTS,S/AS02A	Hepatitis B or pneumococcal conjugate/*H influenzae* type B	ACDi, blood films every 2 weeks for 9 months then passive only	36 months (24–45)	70	22%	45% (31 to 56)
Bagamoyo, Tanzania^13^	209	RTS,S/AS01E	Placebo	PCD	1·8 months (1·7–1·9)	30	Not recorded	53% (26 to 70)
Gabon^13^	215	RTS,S/AS01E	Placebo	PCD	1·5 months (1·4–1·7)	5	Not recorded	53% (26 to 70)
Ghana^13^	81	RTS,S/AS01E	Placebo	PCD	1·6 months (1·5–1·8)	80	Not recorded	53% (26 to 70)
Kilifi, Kenya^12^, ^15^	447	RTS,S/AS01E	Rabies	ACDc, weekly visits	11 months (8–14)	35	59%	53% (28 to 69)
Korogwe, Tanzania^12^, ^15^	447	RTS,S/AS01E	Rabies	ACDc, weekly visits	12 months (9–15)	15	52%	53% (28 to 69)
Kisumu, Kenya^6^	250	RTS,S/AS02A and RTS,S/AS01B	Rabies	ACDi, weekly blood films	25 years (21–29)	60	0%	30% (−15 to 57)
Mozambique (infants)^9^	214	RTS,S/AS02D	Hepatitis B	ACDi, blood films every 2 weeks	1·8 months (1·8–2·1)	45	100%†	66% (43 to 80)
Bagamoyo, Tanzania (infants)^11^	340	RTS,S/AS02D	Hepatitis B	ACDi, blood films every 2 weeks	1·9 months (1·8–2)	30	100%†	65% (21 to 85)

ACDi=active case detection for infection. ACDc=active case detection for clinical malaria. PCD=passive case detection for clinical malaria.

* Age-corrected parasite prevalence data taken from the Malaria Atlas Project.

† Bednets were distributed to every child taking part in these trials and 100% use is assumed.

In the identified trials, healthy adults or children were recruited after clinical and laboratory screening to exclude participants with clinically significant disease. Five trials of children or adults used active case detection for *Plasmodium falciparum* infection (ACDi),^6^, ^8^, ^9^, ^10^, ^11^ one used active case detection for clinical malaria (ACDc),^12^ and two used passive case detection (PCD) for clinical malaria^10^, ^13^ (one trial used both ACDi and PCD). Trials using ACDi and ACDc included assessment of participants who presented with acute illness between scheduled visits, which is usually referred to as PCD in protocols. For simplicity, in our analysis here we use ACDi to refer to the combination of ACDi and PCD, ACDc to refer to combined ACDc and PCD, and PCD to refer to exclusive use of PCD. ACDi was done after antimalarial treatment during the vaccination course and was then monitored with regular finger-prick blood smears. Deaths and severe malaria episodes were monitored in all five trials in which children were enrolled.

### Procedures

We used four primary endpoints in our analysis: infection, clinical malaria, severe malaria, and death. In the trials we identified, infection was defined as any detectable *P falciparum* parasitaemia, with or without a measured fever. We defined clinical malaria as the presence of 2500 or more *P falciparum* parasites per μL of blood in association with reported or measured fever (≥37·5°C).^17^, ^18^ We deemed clinical malaria episodes occurring within 28 days of a previous episode to be part of the same episode. We did not censor time of monitoring according to antimalarial drug use or reported absences from the study area.

We analysed episodes of infection identified by ACDi as one dataset. We combined clinical malaria episodes identified by ACDc and PCD and analysed these as a second dataset. ACDc and PCD were included in the initial study protocols, except for one trial,^13^ in which the efficacy assessment was included after a protocol amendment as an exploratory objective, for which some data were extracted retrospectively.

We identified episodes of severe malaria from safety data reporting. Criteria for severe malaria were derived from the WHO definition^19^ and were applied by the clinical investigators at every site, comprising asexual *P falciparum* parasitaemia, no alternative (or more probable) cause of illness, and either severe malaria anaemia (haemoglobin <50 g/L), cerebral malaria (Blantyre coma score <2), or another symptom (multiple generalised convulsions in 24 h, prostration, hypoglycaemia [<2·2 mmol/L], acidosis, or shock).

Our primary analysis was vaccine efficacy, which was assessed per-protocol. Hence, cohorts monitored for infection or clinical malaria included all participants who had received three doses of vaccine, from 2 weeks after the third dose. We did not judge adults at risk of severe disease or death from malaria. Analysis of severe malaria or death was based on intention to treat and included all children who received at least one dose of vaccine, from the time of the first vaccination.

In Mozambique,^10^, ^14^, ^16^ one cohort (cohort 2) first underwent ACDi in a double-blind phase then subsequently underwent PCD for clinical malaria in a single-blind phase. We included data from the double-blind phase in the ACDi dataset and those from the single-blind phase in the clinical malaria dataset, taking the start of the single-blind phase as the initial time of monitoring for clinical malaria.

We recorded the following covariates across the seven trials: sex, age at the time of vaccination, country, bednet use, adjuvant used (ie, AS01 *vs* AS02), and clinical disease surveillance method at the site level (ie, ACDc *vs* PCD only). To ascertain transmission intensity, we used estimates from the Malaria Atlas Project (MAP) for prevalence of asymptomatic parasitaemia in children aged 2–10 years in 2007 (PrP_2–10_),^20^ identified with the geopositioning coordinates of the trial sites. We refer to this measure here as the local parasite prevalence.

### Statistical analysis

We summarised unadjusted vaccine efficacy for the four endpoints of infection, clinical malaria, severe malaria, and death with Kaplan-Meier curves and efficacy estimates with unadjusted Cox proportional-hazard models for first or only event. To analyse ACDi and combined ACDc and PCD, we assessed the effect of covariates with adjusted Cox proportional-hazard models. We analysed multiple episodes of clinical malaria with Poisson regression, adjusting for the time of follow-up as an offset variable, implemented as one observation per participant.

Rather than present subgroup analyses according to strata (which would be necessarily narrow and be typically confounded by other important covariates), we pooled individual participant data and estimated the linear and non-linear effect of covariates in the data and the interactions of these covariates with vaccine efficacy. We used these empirically observed functions to estimate efficacy in subgroups by multiplying the fixed effect of vaccination (ie, the estimate of the effect of vaccination among those with the baseline value of the covariate) by the interaction term (ie, the estimate of how vaccine efficacy varies for each different level of the covariate). We added variances and the covariance matrix to calculate SEs. All covariates were included in an initial model, and we excluded covariates or interaction terms with p greater than 0·05 to produce a final model. To examine the possibility that analyses of clinical malaria risk were biased by unequal durations of follow-up in some subgroups, we did an additional analysis restricted to 1 year of follow-up.

We calculated vaccine efficacy as either 1 minus the hazard ratio or 1 minus the incidence rate ratio. We modelled the non-linear effects of age at vaccination and local parasite prevalence as multiple fractional polynomials, according to the method of Royston and colleagues.^21^ We fitted changes in vaccine efficacy over time as an interaction between time and vaccination status in Cox regression models, using the Anderson Gill modification,^22^ with clustering by individual to include multiple episodes.

We examined parametric survival models to fit a γ distribution to the unmeasured heterogeneity in exposure. We used a Gompertz survival distribution for parametric models, since this method fitted the data better than the alternatives (exponential, log normal, or Weibull) and gave hazard ratios for vaccination that were nearly identical to those estimated using Cox proportional-hazards.

### Role of the funding source

We did this pooled analysis after a call for proposals initiated and facilitated by GlaxoSmithKline Biologicals. Employees of GSK Biologicals were investigators on the original phase 2 studies and were authors on the primary reports. Employees of GSK Biologicals reviewed the analysis plan and commented on early drafts of the pooled analysis, but were not required to give final approval of the manuscript. The corresponding author had full access to all the data in the study and had final responsibility for the decision to submit for publication.

## Results

We analysed pooled data for 4453 participants in seven trials (table 1). 1376 participants received all three vaccinations, were given curative antimalarial treatment, and underwent ACDi. 3184 participants received all three vaccinations and were monitored for episodes of clinical malaria, either by ACDc or PCD. 465 adults received one or more vaccination; these data were excluded from the analysis for severe malaria or death. 3988 children (ie, younger than 6 years at vaccination) received one or more vaccination and were included in intention-to-treat analyses for severe malaria or death.

**Figure 1:**
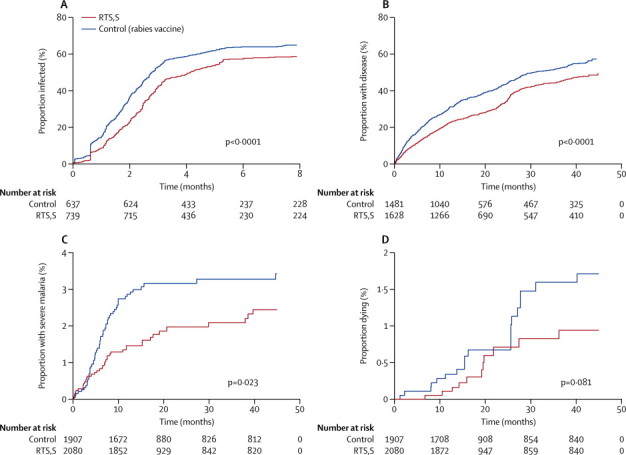
Kaplan-Meier survival plots, according to endpoint (A) Active case detection for infection. (B) First episode of clinical malaria on active or passive case detection for clinical malaria. (C) Severe malaria. (D) Death.

The survival functions for the four endpoints are shown in figure 1. Unadjusted efficacy by ACDi was 33% (95% CI 23–42; p<0·0001). Vaccine efficacy by ACDi did not vary significantly with respect to the covariates tested (table 2). Unadjusted efficacy against clinical malaria by ACDc and PCD was 25% (95% CI 16–33; p<0·0001) for first episodes and 19% (12–25; p<0·0001) for all episodes. However, significant interactions were noted between vaccine efficacy and other covariates (table 2).

**Table 2:** Risk of infection or clinical malaria according to covariate

	**Cox regression for infection (ACDi)**		**Cox regression for first episode of clinical malaria (ACDc/PCD)**		**Poisson regression for all episodes of clinical malaria (ACDc/PCD)**	
	Hazard ratio (95% CI)	p	Hazard ratio (95% CI)	p	Incidence rate ratio (95% CI)	p
RTS,S vaccination	0·78 (0·70–0·88)	0·001	0·63 (0·52–0·77)	<0·0001	0·59 (0·43–0·79)	0·001
Sex (male *vs* female)	1·27 (1·04–1·54)	0·017	1·05 (0·90–1·22)	0·54†	1·05 (0·95–1·15)	0·38†
Sex*RTS,S	1·21 (0·91–1·61)	0·18	0·92 (0·72–1·37)	0·45†	0·99 (0·86–1·14)	0·87†
AS02 trial‡	..	..	1·21 (0·84–1·74)	0·31	1·17 (0·88–1·55)	0·27
AS02 trial*RTS,S	..	..	1·58 (1·01–2·47)	0·046	2·30 (1·54–3·44)	<0·0001
Age (3 years *vs* 5 months)	1·00 (0·99–1·01)	0·81	0·80 (0·76–0·85)	<0·0001	0·79 (0·75–0·84)§	<0·0001
Age*RTS,S	1·00 (0·99–1·01)	0·89†	1·03 (0·92–1·16)	0·62†	0·92 (0·85–0·99)§	0·038
Parasite prevalence (50% *vs* 10%)¶	21·2 (10·3–44)	<0·0001	2·71 (1·54–4·75)	0·001	3·42 (2·35–4·97)§	<0·0001
Parasite prevalence*RTS,S	2·1 (0·72–6·3)	0·17†	2·65 (1·21–5·80)	0·015	2·47 (1·45–4·21)§	0·001
Bednet	0·88 (0·65–1·17)	0·37†	0*·*80 (0*·*63–1*·*01)	0*·*065†	1·08 (0·94–1·24)	0·26†
Bednet*RTS,S	1·07 (0·71–1·62)	0·74†	1·35 (0·96–1·91)	0·064†	0·93 (0·76–1·14)	0·50†
Passive case detection	..	..	0·99 (0·71–1·39)	0·96	0·66 (0·51–0·84)	0·001
Passive case detection*RTS,S	..	..	0·61 (0·38–0·97)	0·039	0·49 (0·34–0·71)	<0·0001

* RTS,S denotes the interaction between RTS,S vaccination and the preceeding covariate. A value of 1 indicates no interaction, with RTS,S having the same effect irrespective of variation in the covariate. A value >1 indicates RTS,S is less effective with the covariate, and a value <1 indicates RTS,S is more effective with the covariate.

† Data were non-significant and therefore were excluded in the final model.

‡ Participants who were randomised in trials of RTS,S/AS02 versus control vaccination.

§ For Cox regression, non-linear effects did not differ from linear effects (p=0·38 and p=0·21, respectively), and hazard ratios refer to linear effects.

¶ Standardised local parasite prevalence in the community in children aged 2–10 years in 2007, derived from the Malaria Atlas Project. Background parasite prevalence and age have been scaled so that the fixed effect of RTS,S is for 20% parasite prevalence at age 1 year.

**Figure 2:**
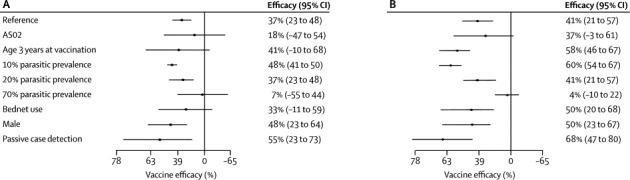
Adjusted forest plots for estimates of vaccine efficacy Reference is RTS,S/AS01, young children (age 12 months), female sex, no bednet use, and low transmission (20% parasite prevalence). (A) First episode of clinical malaria (Cox regression). (B) Multiple episodes of clinical malaria (Poisson regression).

**Figure 3:**
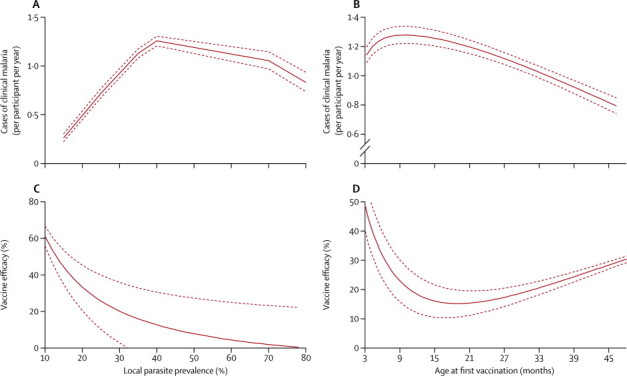
Risk of clinical malaria, according to covariate (A) Incidence of malaria, by local parasite prevalence. (B) Incidence of malaria, by age (months) at vaccination. (C) Vaccine efficacy, by local parasite prevalence. (D) Vaccine efficacy, by age (months) at vaccination. p<0·0001 for non-linear trends shown for multiple fractional polynomials compared with linear trends. Solid line represents the point estimate for efficacy, dotted lines represent the 95% CI.

On Cox regression, vaccine efficacy against first episodes of clinical malaria was 37% (95% CI 23 to 48) with a local parasite prevalence of 20% (moderate transmission; table 2). However, estimated efficacies were 48% (41 to 50) at a local parasite prevalence of 10% (low transmission) and 7% (−55 to 44) at a local parasite prevalence of 70% (high transmission; figure 2). Vaccine efficacy against all episodes of clinical malaria on Poisson regression, allowing for the non-linear effects shown in figure 3, was 41% (95% CI 21 to 57) at a local parasite prevalence of 20% (table 2). Estimated vaccine efficacies were 60% (54 to 67) at a local parasite prevalence of 10% and 4% (−10 to 22) at a local parasite prevalence of 70% (figure 2). Vaccine efficacy also varied by adjuvant choice—eg, at low transmission (PrP_2–10_ 10%) efficacy varied from 60% (95% CI 54 to 67) for AS01 versus 47% (14 to 75) for AS02; however, efficacy did not differ by bednet use or by gender. Vaccine efficacy varied significantly by age for all episodes of clinical malaria (p=0·038), but not for first episodes of clinical malaria (p=0·62; table 2).

Repeating the analysis but restricting follow-up to the first year after vaccination resulted in a similar pattern of results to those reported in table 2, albeit with wider CIs and more marginal significance. For the interaction with adjuvant, the hazard ratio was 1·46 (95% CI 1·00–2·12, p=0·049) and the incidence rate ratio was 2·22 (1·35–3·64). For the interaction with local parasite prevalence, the hazard ratio was 1·51 (0·61–3·72, p=0·38) and the incidence rate ratio was 1·89 (0·93–3·8, p=0·078).

Unadjusted efficacy against severe malaria was 37% (95% CI 6 to 58, p=0·023); data were from 39 children with severe malaria from a total of 2080 RTS,S vaccinated people, versus 58 children with severe malaria from a total of 1908 controls. Efficacy against death was 48% (−8 to 75, p=0·081); 11 deaths occurred in the 2080 people receiving vaccine and 19 deaths happened among the 1908 controls. We judged the frequency of severe malaria and death to be too low to justify further multivariable analysis.

**Figure 4:**
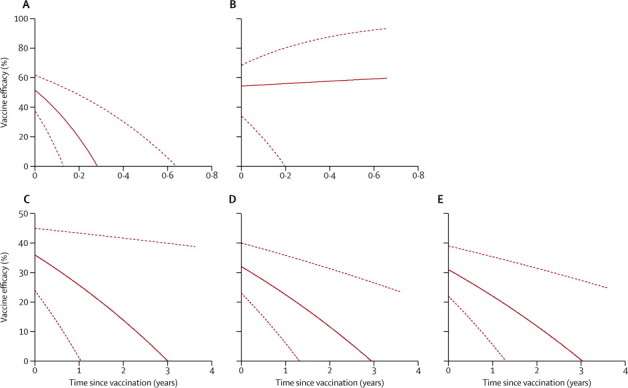
Vaccine efficacy against time (A) Infection on active case detection. (B) Infection on active case detection after adjustment for known variation in exposure to malaria (using local parasite prevalence as a fixed effect) and unknown variation in exposure (fitting a shared γ-distributed frailty). (C) First episodes of clinical malaria on active or passive case detection. (D) Multiple episodes of clinical malaria on active or passive case detection. (E) Multiple episodes of clinical malaria after adjustment for known variation in exposure to malaria (using local parasite prevalence as a fixed effect) and unknown variation in exposure (fitting a shared γ-distributed frailty). Solid line represents the point estimate for efficacy, dotted lines represent the 95% CI.

The survival plot of time to infection during ACDi by vaccination status shows convergence after the initial divergence (figure 1A), and the plot of time to clinical malaria by ACDc and PCD shows a gradual slowing in the rate of divergence (figure 1B). An interaction between efficacy and time gave similar goodness of fit (judged by Akaike's information criterion) for various powers of time (2, −1, −2, 0·5, 0·25) and linear and log functions. We therefore selected a linear fit for simplicity and to make interpretation of the interaction terms more intuitive (figure 4).

In unadjusted analysis of ACDi, efficacy seems to wane rapidly (figure 4A), but after adjustment for local parasite prevalence and for a γ-distributed shared frailty (θ=0·96, p<0·0001, indicating that significant evidence exists for pronounced heterogeneity of risk), the effect of vaccination did not vary by much over time (figure 4B, table 3). In unadjusted and adjusted analyses of ACDc and PCD, including single and multiple clinical episodes, the estimated vaccine efficacy fell over time, from 36% efficacy (95% CI 24 to 45) at time of vaccination to 0% (95% CI −38 to 38) at 3 years (figures 4C–E).

**Table 3:** Vaccine efficacy over time

	**Fixed effect (or constant term) for vaccination with RTS,S**		**Interaction between RTS,S and time (years)**	
	Hazard ratio (95% CI)	p	Hazard ratio (95% CI)	p
ACDi	0·48 (0·38 to 0·62)	<0·0001	13·1 (4·5 to 38)	<0·0001
ACDi, adjusted*	0·46 (0·32 to 0·66)	<0·0001	0·83 (0·1 to 8·1)	0·79
Clinical malaria, single episodes	0·64 (0·55 to 0·76)	<0·0001	1·16 (1·03 to 1·3)	0·016
Clinical malaria, multiple episodes	0·68 (0·60 to 0·77)	<0·0001	1·14 (1·07 to 1·22)	<0·0001
Clinical malaria, adjusted*	0·69 (0·61 to 0·78)	<0·0001	1·13 (1·06 to 1·21)	<0·0001

Every row represent coefficients from a single model. The fixed effect of vaccination reflects the hazard ratio associated with vaccination at 0 years. The interaction term reflects the change in hazard ratio associated with every year since vaccination.

* Transmission intensity is a fixed effect to account for known variation in exposure to malaria, and γ-distributed shared frailty accounts for unknown variation in exposure to malaria. ACDi=active case detection for infection.

We tested for heterogeneity in the rate of declining vaccine efficacy against clinical malaria by estimating the three-way interactions between time (in years), vaccination, and every covariate in turn. None of these three-way interaction terms were significant at the 5% level (AS02 *vs* AS01, hazard ratio 0·94, 95% CI 0·82–1·09, p=0·46; local parasite prevalence hazard ratio 1·35, 0·74–2·46, p=0·33; age in years hazard ratio 0·97, 0·93–1·01, p=0·15).

## Discussion

The findings of our pooled analysis show that the RTS,S malaria vaccine is protective against infection and disease. However, unadjusted efficacy against clinical malaria was lower than previous estimates in children age 5–17 months^12^ and substantial heterogeneity was noted in efficacy between population subgroups and over time. Vaccine efficacy against clinical malaria was lowest at high (70%) transmission intensity, and it was reduced for the AS02 adjuvant compared with AS01. Weak variation in efficacy was noted according to age on Poisson regression, which was not significant on Cox regression. Vaccine efficacy did not vary by gender or bednet use. Results for efficacy from Cox regression for first episodes and Poisson regression for all episodes were similar, although CIs suggested greater precision when all episodes were included.

A higher vaccine efficacy with PCD versus ACDc might indicate bias resulting from a prophylactic effect of antimalarial drugs administered for episodes of malaria that do not meet the case definition. These malaria episodes are likely to be more common in unvaccinated children and hence could result in an underestimate of vaccine efficacy on ACDc. However, no sites used PCD and ACDc alongside each other, hence there is confounding by site and the difference might reflect other variations between sites that were not measured by the available covariates. To examine whether additional unmeasured factors that segregate by site might lead to varying efficacy, we fitted a post-hoc interaction term between vaccination and stratification by site, in addition to the previous model (table 2). These additional interactions significantly improved model fit (p<0·0001, by likelihood-ratio test), indicating that other unmeasured factors cause vaccine efficacy to vary between sites.

Transmission intensity (as measured by local parasite prevalence in children age 2–10 years) had a non-linear effect on clinical malaria incidence.^23^ The incidence of clinical malaria reached a peak in areas with a local parasite prevalence of 40%. This finding could be accounted for by children who acquire greater immunity with increasing exposure, which offsets the rises in incidence of clinical malaria that otherwise might be seen at a higher local parasite prevalence.

RTS,S can be regarded as a leaky barrier to infection, because it protects against some infectious bites but not against others.^24^ The probability of protecting a participant exposed to two infective bites during the course of a night against a subsequent episode of clinical malaria is half the probability of protecting a participant exposed to one bite. This statistic suggests that vaccine efficacy will be lower at high transmission intensity, which accords with our observations.

We used MAP estimates of age-adjusted prevalence of asymptomatic malaria (PrP_2–10_) to gauge transmission intensity. These approximations were based on several thousand surveys in the countries where trial sites were located. We chose these standardised independent measures rather than within-trial factors, such as incidence of malaria among controls, because monitoring was not the same between trials. MAP estimates do not account for changes over time, but transmission intensity at our sites is likely to be stable enough over a few years for these data to be a reasonable approximation. Variations in seasonal transmission have a modest effect on the relation between entomological inoculation rate and asymptomatic parasitaemia, but in view of the limitations of using data from 11 sites, we did not feel that more complex characterisations of transmission intensity were warranted.

Our finding that vaccine efficacy is not affected by use of insecticide-treated bednets but is diminished at higher levels of transmission intensity might seem contradictory, since bednet use might be expected to reduce exposure and, hence, enhance vaccine efficacy. However, individual use of insecticide-treated bednets might be only modestly protective (compared with greater mass effects at reducing transmission when whole communities use bednets). Furthermore use of insecticide-treated bednets was not distributed evenly by site, varying from 4·5% to 100% of children. Tests for variation in vaccine efficacy by bednet use are, therefore, vulnerable to ecological confounding by site.

We identified significant interactions between time and vaccine efficacy. We can confidently reject the null hypothesis that vaccine efficacy is constant over time (p<0·0001), but we cannot be confident about the shape of the plotted decline, which is reflected in the wide CIs surrounding estimates of efficacy at later timepoints (figure 4). We chose a linear interaction for simplicity of presentation, although power functions of time fit the data slightly better. Therefore, we cannot extrapolate beyond the data to longer durations of follow-up, since the shape of the line is determined by statistical convenience rather than biological understanding.

A fall in vaccine efficacy over time might be attributable to systematic bias in estimates obtained using survival analysis because of heterogeneous exposure from a partly effective vaccine, as previously described.^25^ Comparison of figure 4A with figure 4B suggests that systematic bias resulting from heterogeneous exposure can account for the apparent waning of efficacy in the ACDi dataset, rather than a genuine biological waning of efficacy taking place. However, similarity between figures 4C–E suggests that no clear systematic bias exists in estimates of efficacy over time when using data from ACDc or PCD for clinical malaria. The difference could be because ACDi was monitored during one transmission season, with only 60% of participants having an episode during this period. A few unexposed individuals can lead to a biased estimate of rapidly declining efficacy.^25^ However, data for clinical malaria included 4 years of monitoring during many transmission seasons. Furthermore, individual exposure could vary from year to year.^26^ Hence, a discrete unexposed population is less likely to exist with ACDc or PCD compared with ACDi. Heterogeneous exposure, therefore, seems to be a sufficient explanation for the observation that efficacy wanes more rapidly in the ACDi dataset than it does in the ACDc and PCD dataset.

We report here all phase 2 data for RTS,S malaria vaccines (panel), including efficacy outcomes for clinical malaria (ACDc and PCD) and for malaria infection (ACDi). Some phase 2 trials also included cross-sectional surveys for asymptomatic parasitaemia. In the Mozambique trial, substantial protection against asymptomatic parasitaemia was noted 45 months after vaccination in cohort 1,^14^ which is longer than would have been predicted by our analysis. On the other hand, protection against asymptomatic parasitaemia was not noted in Mozambique cohort 2 at 21 months after vaccination. This finding could be explained by differential acquisition of blood-stage immunity between these two cohorts.^27^ Our data do not allow us to distinguish waning vaccine-induced immunity from delayed acquisition of blood-stage immunity, but analysis of the effect of the booster vaccination—planned as part of the phase 3 trial—is likely to be highly informative. A booster dose can restore vaccine-induced immunity but will not have an immediate effect on immunity to blood-stage parasites. Furthermore, the larger sample size in the phase 3 trial will provide more accurate point estimates for efficacy in the age-groups assessed (ie, 6–12 weeks and 5–17 months) than is possible in a meta-analysis of phase 2b data.

Panel **Research in context**

**Systematic review**

We searched PubMed with the MeSH terms “RTS,S-AS02D vaccine [Substance Name]” OR “RTS,S-AS01E vaccine [Substance Name]” OR “RTS,S-AS01B vaccine [Substance Name]” OR “RTS,S-AS02A vaccine [Substance Name]” OR “PfCSP DNA vaccine [Substance Name]” AND “Malaria, falciparum/prevention and control”. All phase 2 trials identified are included in our analysis and no previous meta-analyses have been done. Two publications describe preliminary data from phase 3 trials, for which follow-up is ongoing. These preliminary reports have not reported subgroup analyses, and data were not available for inclusion in our analysis.

**Interpretation**

Vaccine efficacy varied by transmission intensity, adjuvant, and time since vaccination. Age at first vaccination was of borderline significance, but bednet use or sex had no effect on efficacy. Vaccine efficacy against clinical disease was of limited duration and fell with increasing transmission intensity. Outcomes after vaccination cannot be predicted accurately on the basis of one pooled efficacy figure. When predicting public health outcomes of vaccination, variations in efficacy—by transmission intensity and by time since vaccination—will need to be accounted for.

In summary, we noted significant variation in estimated vaccine efficacy by population subgroups and a significant decline in protection against clinical malaria over time. One might argue that the unadjusted pooled estimates of efficacy nevertheless reflect what was actually seen in the population tested. However, the unadjusted pooled efficacy is merely a weighted mean of efficacies seen in the component subgroups of the population and, therefore, cannot be generalised to other populations. For instance, if the vaccine is more effective at lower transmission intensity, the pooled vaccine efficacy will depend on the proportion of children recruited in sites at low transmission intensity. Predictions of public health outcomes of vaccination will need to take account of these variations in efficacy by transmission intensity and by time since vaccination.

## References

[1] Ceesay SJ, Casals-Pascual C, Erskine J (2008). Changes in malaria indices between 1999 and 2007 in The Gambia: a retrospective analysis. Lancet.

[2] O'Meara WP, Bejon P, Mwangi TW (2008). Effect of a fall in malaria transmission on morbidity and mortality in Kilifi, Kenya. Lancet.

[3] The RTS,S Clinical Trials Partnership (2011). First results of phase 3 trial of RTS,S/AS01 malaria vaccine in African children. N Engl J Med.

[4] Moorthy VS, Ballou WR (2009). Immunological mechanisms underlying protection mediated by RTS,S: a review of the available data. Malar J.

[5] Kester KE, Cummings JF, Ofori-Anyinam O (2009). Randomized, double-blind, phase 2a trial of falciparum malaria vaccines RTS,S/AS01B and RTS,S/AS02A in malaria-naive adults: safety, efficacy, and immunologic associates of protection. J Infect Dis.

[6] Polhemus ME, Remich SA, Ogutu BR (2009). Evaluation of RTS,S/AS02A and RTS,S/AS01B in adults in a high malaria transmission area. PLoS One.

[7] Halloran ME, Haber M, Longini IM (1992). Interpretation and estimation of vaccine efficacy under heterogeneity. Am J Epidemiol.

[8] Bojang KA, Milligan PJ, Pinder M (2001). Efficacy of RTS,S/AS02 malaria vaccine against *Plasmodium falciparum* infection in semi-immune adult men in The Gambia: a randomised trial. Lancet.

[9] Aponte JJ, Aide P, Renom M (2007). Safety of the RTS,S/AS02D candidate malaria vaccine in infants living in a highly endemic area of Mozambique: a double blind randomised controlled phase I/IIb trial. Lancet.

[10] Alonso PL, Sacarlal J, Aponte JJ (2004). Efficacy of the RTS,S/AS02A vaccine against *Plasmodium falciparum* infection and disease in young African children: randomised controlled trial. Lancet.

[11] Abdulla S, Oberholzer R, Juma O (2008). Safety and immunogenicity of RTS,S/AS02D malaria vaccine in infants. N Engl J Med.

[12] Bejon P, Lusingu J, Olotu A (2008). Efficacy of RTS,S/AS01E vaccine against malaria in children 5 to 17 months of age. N Engl J Med.

[13] Asante KP, Abdulla S, Agnandji S (2011). Safety and efficacy of the RTS,S/AS01_E_ candidate malaria vaccine given with expanded-programme-on-immunisation vaccines: 19 month follow-up of a randomised, open-label, phase 2 trial. Lancet Infect Dis.

[14] Sacarlal J, Aide P, Aponte JJ (2009). Long-term safety and efficacy of the RTS,S/AS02A malaria vaccine in Mozambican children. J Infect Dis.

[15] Olotu A, Lusingu J, Leach A (2011). Efficacy of RTS,S/AS01E malaria vaccine and exploratory analysis on anti-circumsporozoite antibody titres and protection in children aged 5–17 months in Kenya and Tanzania: a randomised controlled trial. Lancet Infect Dis.

[16] Alonso PL, Sacarlal J, Aponte JJ (2005). Duration of protection with RTS,S/AS02A malaria vaccine in prevention of *Plasmodium falciparum* disease in Mozambican children: single-blind extended follow-up of a randomised controlled trial. Lancet.

[17] Olotu A, Fegan G, Williams TN (2010). Defining clinical malaria: the specificity and incidence of endpoints from active and passive surveillance of children in rural Kenya. PLoS One.

[18] Mabunda S, Aponte JJ, Tiago A, Alonso P (2009). A country-wide malaria survey in Mozambique: II—malaria attributable proportion of fever and establishment of malaria case definition in children across different epidemiological settings. Malar J.

[19] WHO (2004). Management of severe malaria: a practical handbook.

[20] Hay SI, Guerra CA, Gething PW (2009). A world malaria map: *Plasmodium falciparum* endemicity in 2007. PLoS Med.

[21] Royston P, Reitz M, Atzpodien J (2006). An approach to estimating prognosis using fractional polynomials in metastatic renal carcinoma. Br J Cancer.

[22] Andersen PK, Gill RD (1982). Cox's regression model for counting processes: a large sample study. Ann Stat.

[23] Snow RW, Omumbo JA, Lowe B (1997). Relation between severe malaria morbidity in children and level of *Plasmodium falciparum* transmission in Africa. Lancet.

[24] Maire N, Aponte JJ, Ross A (2006). Modeling a field trial of the RTS,S/AS02A malaria vaccine. Am J Trop Med Hyg.

[25] White MT, Griffin JT, Drakeley CJ, Ghani AC (2010). Heterogeneity in malaria exposure and vaccine response: implications for the interpretation of vaccine efficacy trials. Malar J.

[26] Bejon P, Williams TN, Liljander A (2010). Stable and unstable malaria hotspots in longitudinal cohort studies in Kenya. PLoS Med.

[27] Guinovart C, Aponte JJ, Sacarlal J (2009). Insights into long-lasting protection induced by RTS,S/AS02A malaria vaccine: further results from a phase IIb trial in Mozambican children. PLoS One.

